# Muscle coordination and recruitment during squat assistance using a robotic ankle–foot exoskeleton

**DOI:** 10.1038/s41598-023-28229-4

**Published:** 2023-01-24

**Authors:** Hyeongkeun Jeong, Parian Haghighat, Prakyath Kantharaju, Michael Jacobson, Heejin Jeong, Myunghee Kim

**Affiliations:** 1 Department of Mechanical and Industrial Engineering, University of Illinois at Chicago, Chicago, IL 60607 USA; 2 Ira A. Fulton Schools of Engineering, Arizona State University, Arizona, Mesa, AZ 85212 USA

**Keywords:** Biomedical engineering, Mechanical engineering

## Abstract

Squatting is an intensive activity routinely performed in the workplace to lift and lower loads. The effort to perform a squat can decrease using an exoskeleton that considers individual worker’s differences and assists them with a customized solution, namely, personalized assistance. Designing such an exoskeleton could be improved by understanding how the user’s muscle activity changes when assistance is provided. This study investigated the change in the muscle recruitment and activation pattern when personalized assistance was provided. The personalized assistance was provided by an ankle–foot exoskeleton during squatting and we compared its effect with that of the no-device and unpowered exoskeleton conditions using previously collected data. We identified four main muscle recruitment strategies across ten participants. One of the strategies mainly used quadriceps muscles, and the activation level corresponding to the strategy was reduced under exoskeleton assistance compared to the no-device and unpowered conditions. These quadriceps dominant synergy and rectus femoris activations showed reasonable correlations (r = 0.65, 0.59) to the metabolic cost of squatting. These results indicate that the assistance helped reduce quadriceps activation, and thus, the metabolic cost of squatting. These outcomes suggest that the muscle recruitment and activation patterns could be used to design an exoskeleton and training methods.

## Introduction

Fatigue is one of the most prevalent symptoms experienced by workers on a daily basis, affecting 54% of individuals^1^ and resulting in reduced productivity and increased health care costs^2,3^. Muscular fatigue is a major risk factor, among risks such as mental, visual, and auditory fatigue^4^. For example, quadriceps muscle fatigue changes the posture during a squat and increases the load on the lower back, resulting in an increased risk of lumbar injury^5,6^. An intervention that reduces such fatigue would help improve productivity and reduce the risk of injury. This intervention can be provided by assistive technology, such as exoskeletons^7^, as these devices can augment workers’ performance, relieve the physical workload of standing^8–10^ and squatting^11–15^, and reduce fatigue^5^. Assistive technology has been found to be more effective when it is tuned for each user^16,17^. Similarly, our prior squat assistance study found that a robotic ankle–foot exoskeleton can help reduce physical effort, measured by metabolic cost, especially when tuned for each user^18^. Considering the correlation between fatigue and physical effort^19^, the personalized device can be helpful; however, the mechanism by which physical effort is reduced remains unclear.

The mechanism underlying the reduction in metabolic cost with squat assistance may be understood by investigating the role of muscle recruitment patterns. Physical effort, measured by metabolic cost, appears to be correlated with muscular activity^20,21^, and muscle recruitment and coordination patterns^22^. Researchers also found that dominant muscles could help to estimate the metabolic cost^23^. Muscle synergy analysis is a computational approach used to quantify coordination patterns during dynamic tasks^24–26^. This method is based on the theory that a simple movement involves numerous and redundant muscle groups acted on by the central nervous system (CNS). Yet, this theory remains unclear how the system overcomes this complexity and adopts an optimal recruitment strategy^27^. The muscle synergy approach posits that the CNS simplifies its decision-making and organizes movements by grouping functionally similar muscles into modules (called muscle synergies) and coordinating them as a single unit. The modules rather than individual muscles^27–29^ perform the action, which results in dimensional reduction^27,30^.

Muscle synergy analysis calculates muscle recruitment and activation patterns from electromyography (EMG) data^31,32^ by decomposing EMG activation patterns into a reduced dimension of time-varying signals and a matrix of weights, or synergy vectors. The synergy signal and weight can be linearly combined to reconstruct the original EMG signals. Studies found that a small set of synergies can explain over 95% of the muscle activity across tasks such as walking, running, or high stepping^24,33–35^, and consistent muscle synergies exist between subjects^36–38^ across the experiment time^39,40^. Researchers investigated the effect of outside factors on common synergies, such as exoskeletons, inclination, or gait speed^41,42^. We hypothesize that a common muscle coordination pattern would exist across participants during a squat, and the activation levels of the coordination would be correlated with the metabolic rate. Also, prior findings on subject-specific muscle synergy could be used to help understand personalized assistance^42^.

**Figure 1:**
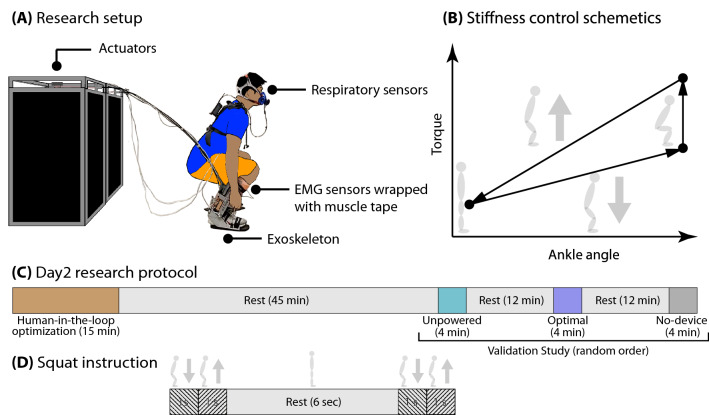
Experiment overview. (**A**) Experimental setup: subjects wore an ankle–foot exoskeleton end-effector and a respiratory sensor. EMG sensors were attached at eight positions. The power and control signals were transmitted from the actuators to the exoskeleton end-effector via Bowden cables. (**B**) Squat controller: the controller used different stiffness parameters depending on the squat phases. (**C**) Experimental protocol: personalized stiffness parameters were identified using the human-in-the-loop optimization and evaluated during a validation study. We investigated data from the validation study. (**D**) For each squat, subjects were instructed to perform a 2-s squat and 6-s rest.

This research aims to evaluate the change in muscle activity and recruitment patterns in 10 participants who performed squatting with an ankle–foot exoskeleton that provided personalized assistance (Fig. 1). We used muscle activity and respiratory rate data during the validation trials. The conditions tested were (1) no exoskeleton, (2) the personalized assistance condition (optimal condition), and (3) the unpowered exoskeleton condition (Fig. 1C). We tested the hypotheses by investigating each muscle activation, the number of synergies, synergy similarity after classification, the muscle synergy pattern coordination in a squat cycle, and the correlation between the muscle analysis result and metabolic cost. The outcomes of this research could help inform future exoskeleton design processes.

## Results

### Muscle activation analysis

**Figure 2:**
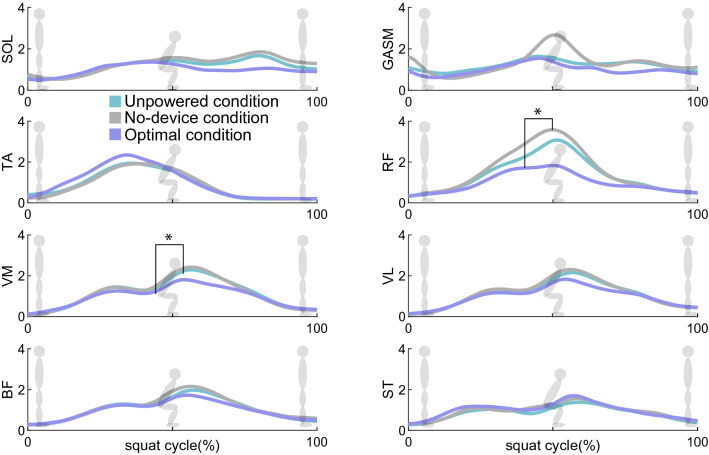
The graphs are the mean trajectories of muscle activations in each condition during squat cycles. 0% is the stance phase, 50% is the bottom point, and 100% is the stand-back point. The blue line is the activation of the optimal condition. The grey line is the no-device condition, and the green line is the unpowered condition trajectory. For statistical expression, * is p < 0.05.

On the assisted leg, the conditions influenced the rectus femoris (RF) and vastus medialis muscle activities (Friedman p = 0.032, Friedman p = 0.016). For the personalized assistance condition, the RF activities were statistically significantly reduced by 25.5% for the optimal condition compared to the no-device condition (p = 0.032) (Fig. 2). The vastus medialis (VM) activity was statistically significantly reduced by 10.6% for the optimal condition compared to the unpowered condition (p = 0.012). The activation of tibialis anterior (TA), soleus (SOL), gastrocnemius medialis (GASM), vastus lateralis (VL), bicep femoris (BF), and semitendinosus (ST) were not statistically significantly affected by the conditions (ANOVA or Friedman, p > 0.054).

During the descent phase of the squat, the conditions affected the RF muscle activities (Friedman p = 0.018). The RF muscle activities were reduced by 24% for the optimal condition, compared to the no-device condition (p = 0.023). Other muscle activation levels were not statistically significantly influenced by conditions (ANOVA or Friedman > 0.052). During the ascent phase of the squat, the assistance influenced RF muscle activities (ANOVA p = 0.004). RF reduced muscle activity levels by 25.4% and 19.3% for the optimal condition compared to the unpowered and no-device conditions (p = 0.037, p = 0.037). The assistance conditions also influenced the SOL muscle activity (ANOVA p = 0.031). However, its pairwise differences were not statistically significant (p = 0.016). Other muscles were not statistically significantly affected by conditions (ANOVA or Friedman p > 0.051).

### Number of synergies

3 to 5 synergies from the assisted leg were identified for all subjects, and 7 subjects showed 4 synergies (percentage of minimum variance accounted for (VAF) between validation study conditions (the no-device, unpowered, and personalized condition)).

### Muscle synergy comparison

**Figure 3:**
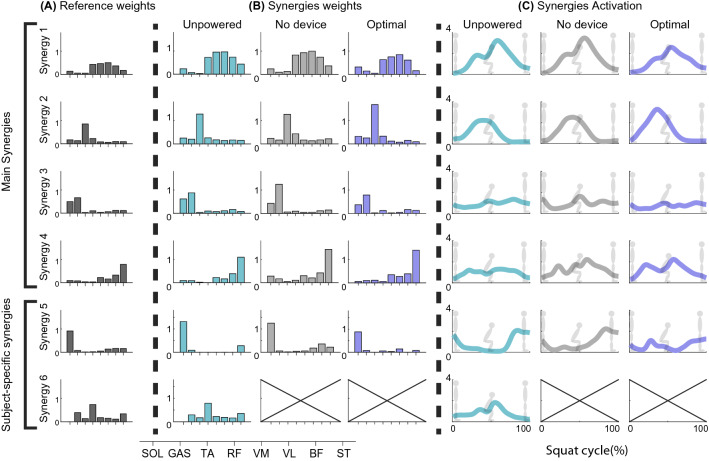
Muscle synergies from the assisted leg. (**A**) Reference weights from the K-means algorithm applied to 10 subjects’ data in all conditions. (**B**) Synergy weights assignment without duplication by condition. (**C**) Synergy activations, mean trajectories of all subjects, of corresponding synergy weights in (**B**).

We identified 4 synergy references (synergy1 – 4) and 2 subject-specific synergies (synergy5, 6, Fig. 3) from the assisted leg. Synergy1 is the quadriceps dominant synergy (inter-subject similarity of all conditions: $0.93\pm 0.04$, Table 1A). This synergy was active during the second half of the descent squat for downward deceleration and ascent squat for upward acceleration^43^. Synergy2 was the tibialis anterior (TA) dominant synergy (inter-subject similarity of all conditions: $0.92\pm 0.08$, Table 1A), active during the first half of the squat phase for accelerating downward movement with dorsiflexion^43^. Synergy3 was the dominant synergy of soleus (SOL) and gastrocnemius medialis (GASM). Synergy3 helped in plantarflexion and was activated to a greater extent in the second half of the squat phase ($0.9\pm 0.06$, Table 1A)^43^. Synergy4 mainly consisted of bicep femoris (BF) and semitendinosus (ST), which reduced shear stress on the knee, and was usually co-activated with synergy1^44^. Synergy1, synergy2, and synergy3 were recruited from most subjects (Table 1B). Two participants presented subject-specific synergy5 for all conditions, and two participants showed subject-specific synergy 6 for the unpowered condition. Synergy5 was soleus (SOL) dominant synergy, and synergy6 was rectus femoris (RF) dominant synergy.

**Table 1:** (A) Similarity scores with the same synergies, in each condition. (B) The number of synergies assigned in each synergy group for a specific condition. The maximum sample number is 9, 10, and 10 for the unpowered, no-device, and optimal condition.

(A) Similarity (mean $\pm$ std)				(B) Assigned number of samples		
	Unpowered	No-device	Optimal	Unpowered	No-device	Optimal
Synergy1	$0.94\pm 0.04$	$0.92\pm 0.03$	$0.94\pm 0.04$	8	10	9
Synergy2	$0.93\pm 0.05$	$0.91\pm 0.10$	$0.94\pm 0.06$	9	9	10
Synergy3	$0.93\pm 0.07$	$0.90\pm 0.07$	$0.91\pm 0.07$	9	10	9
Synergy4	$0.92\pm 0.06$	$0.86\pm 0.10$	$0.94\pm 0.05$	5	6	9

### Synergy activation analysis

**Figure 4:**
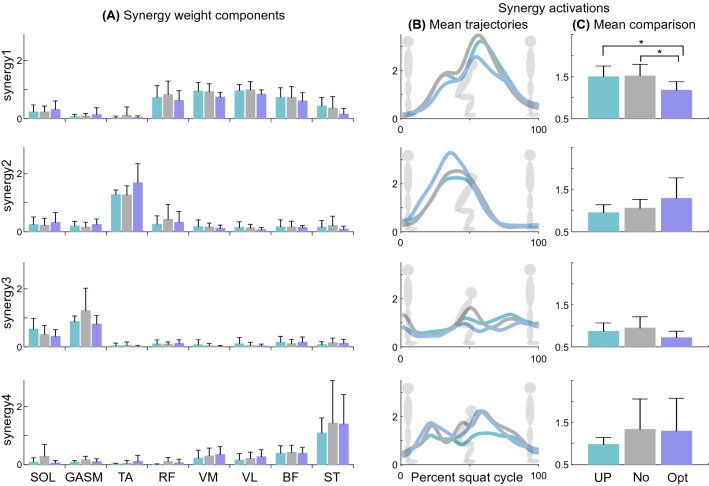
Comparison of 4 main synergies for three different conditions, unpowered (UP), no-device (NO), and optimal (Opt). (**A**) Synergy weight component. (**B**) Synergy activation trajectory through the squat cycle. (**C**) The mean values of synergy activations. Bar graphs show the mean and standard deviation. For statistical expression, * is p < 0.05.

The assistance conditions affected the mean activation level of synergy1, the quadriceps dominant synergy (ANOVA p = 0.004) (Fig. 4A). It was statistically significantly lower by 22.3% and 21.3% (Fig. 4C) for the personalized condition, compared to the no-device condition and unpowered condition, respectively (p = 0.046, p = 0.026). Other synergy activation levels were not statistically significantly influenced by conditions (ANOVA or Friedman p > 0.081).

During the descent phase of the squat, none of the synergy activation levels were statistically significantly affected by the conditions (ANOVA or Friedman p > 0.79). During the ascent phase of the squat, the conditions affected the synergy1 activation level (ANOVA p = 0.002). Synergy1 was reduced by 22.6% for the optimal condition compared to the unpowered condition (p = 0.013). Other synergy activation levels were not statistically significantly influenced by the conditions (ANOVA or Friedman p > 0.217).

### Relation between muscles activation and metabolic cost

The Pearson correlation coefficient between the metabolic cost and RF muscle activity level was 0.59 (p = 0.001). The correlation between metabolic cost and synergy1 activation level was 0.65 (p < 0.001).

## Discussion

This study investigated the effect of squat assistance using a unilateral ankle–foot exoskeleton on muscle activities and their coordination pattern. We hypothesized that the effect of assistance from the device would be reflected in muscle coordination, activation patterns, and muscle synergies. We also hypothesized that shared synergies would exist across subjects, which can be used as an indicator of change in physical effort. We found that the optimal assistance provided by the ankle–foot exoskeleton helped reduce the quadriceps dominant synergy (synergy1) activation compared to the unpowered and no-device conditions. The muscle activation level of the RF muscle was also reduced compared to the no-device condition. We also identified a moderately high and significant correlation^45,46^ between metabolic cost and quadriceps muscle activation level.

The optimal assistance by the ankle–foot exoskeleton reduced the load on quadriceps muscles, including RF, VM, and VL. During the second quarter of a squat cycle (25–50% of the squat cycle), the muscles were activated to decelerate downward squatting. For the third quarter of the cycle (50–75% of the squat cycle), they helped accelerate upward squatting (Fig. 2)^43^. Surprisingly, the ankle assistance reduced the muscle group activation levels compared to the unassisted conditions. The reduction could be explained by the following principles: closed-chain exercise and energy transmission. During closed chain exercise, a limb is fixed while other body parts freely move; hence any motion in one joint in a kinematic chain influences other joints in the chain^47,48^. In our experiment, the squat can be considered a closed-chain exercise where ankle joint assistance affected other joints in the chain, including quadriceps muscle^43,49,50^. Also, the fact that quadriceps muscles were benefited from assistance in the descent and ascent squat phases could be interpreted as the result of closed chain and energy transmission as examined in whole squat phase activation. Prior studies found that when ankle work was restricted, the quadriceps muscle activities increased for jumping activity^51^. This study optimally assisted the ankle, and it appears that the optimal assistance reduced quadriceps muscle activities, especially during the ascent phase of the squat. Since quadriceps muscles mainly experience fatigue during the squat, ankle–foot assistance may help reduce fatigue^52,53^.

The other muscle coordination and activation patterns appear to be maintained under ankle assistance. TA and its dominant synergy activations (synergy2, Figs. 3, 4) were activated during the first half of the squat cycle (0–50% of the squat cycle) for initiating dorsiflexion^43^. They increased during the first half of the squat cycle for the optimal condition. The downward movement also includes the contribution from the knee and hip flexion muscles, especially in the initial phase (0–25% of the squat cycle)^54^. The participants tended to increase the synergy2 activities, suggesting that the participants might not have fully learned how to coordinate their muscle activation patterns to be more efficient. Also, in the study^55^, the optimization was conducted for the entire squat cycle. Phase-specific optimization may help maintain or reduce the activation level of the synergy2. SOL and GASM and their dominant synergy activations (synergy3, Figs. 3, 4), working for plantar flexion, were expected to decrease using the optimal ankle assistance condition. However, the change was not significantly different (p > 0.08) (Figs. 2, 4). This outcome also might be due to an additional effort to stabilize the ankle joint using TA, SOL, and GASM^54,56,57^. Prior studies presented the importance of training to learn the method of using the wearable robot^58^. Additional training may reveal the change in the other muscle coordination and activation patterns.

Our study suggests that the quadriceps dominant synergy could be used to design assistance. Similar to other studies using muscle information to predict metabolic cost during walking^23,59–61^ (correlation of 0.55–0.65^46,62^), our study found a moderately high correlation between the metabolic cost of squatting and the quadriceps muscle synergy (r = 0.65) and also between the metabolic cost and RF activity (r = 0.59). In our study, all subjects did not show quadriceps dominant synergy (synergy1); however, all subjects showed a cosine similarity value higher than 0.86 for synergy1, which can be used to assign a synergy considering that the similarity criteria was 0.8^42^. This result suggests that using RF or quadriceps dominant synergy activation could be an approximate measure of the metabolic cost of squatting.

Squatting posture could influence the magnitude of quadriceps activation^63^. For instance, a previous study showed that mediolateral knee malalignment during the squat could decrease quadriceps activation while increasing hamstring and gastrocnemius activations^63^. In our study, however, we closely monitored our subjects and instructed them regarding their squat depth and posture if needed. We did not observe an increase in the hamstring and gastrocnemius activation. Also, the squat ankle angle calculated from the IMU showed a change of less than 2 degrees across conditions, and the variation was not statistically significant (p > 0.4)^55^. These results suggest that the quadriceps activation decreased due to ankle–foot assistance rather than improper squat posture. To obtain more precise results, future work could include squat posture feedback mechanisms using a real-time motion analysis system during the experiment^58^.

This study investigated the muscle activations and their coordination patterns for different squatting conditions with an assistive exoskeleton. We observed common muscle activation and activation patterns across participants during the squat, and the exoskeleton assistance was found to reduce the quadriceps dominant synergy activation. This reduced activation could help delay fatigue^53^. We also found that the activation level showed a reasonable correlation^45,64^ with the metabolic cost. Further research is needed to optimize the exoskeleton parameters for personalized squat assistance using muscle activation as an objective function (as an alternative to metabolic cost). For future work, we aim to develop practical and comfortable methods by utilizing both quadriceps dominant and TA dominant synergies for human-in-the-loop optimization of the exoskeleton for personalized squat assistance.

## Methods

### Research protocol

Ten unimpaired male subjects (age: $24.6\pm 4$ years, weight: 78.86 $\pm$ 11.30 kg, height: 176.65 ± 8.24 cm (Mean ± std)) participated in the research study. Subjects wore an ankle–foot exoskeleton (developed in our lab)^65^, powered by two off-board actuators (Humotech, Pittsburgh, Pennsylvania, USA), on their dominant leg. The power and signals were transmitted through a tether (Fig. 1A). The subjects participated in a 2-day experimental protocol, where Day 1 was for acclimation day and Day 2 was for human-in-the-loop (HIL) optimization and validation study (Fig. 1C). The detailed procedure is explained in Kantharaju et al.^55^.

The exoskeleton was controlled with two stiffness parameters to tune its assistive torque during the descent and ascent phases of the squat (Fig. 1B). In this paper, we have analyzed the data from the validation study. The muscle activation patterns during the squat with exoskeleton assistance in three different conditions, the no-device, unpowered, and optimal stiffness conditions, were investigated. This research protocol was approved by the University of Illinois at Chicago Institutional Review Board based on the Declaration of Helsinki. All participants (above 17 years) provided written informed consent before the study.

### Acclimation day

This day was provided for participants to experience squat assistance^66–68^. Subjects went through 6 squat conditions for 4 min each: four random assistance conditions with combinations of descending and ascending squat stiffness parameters based on their weights, unpowered exoskeleton, and no-device conditions. The order of conditions was randomly determined^55^ and the rest times of 12 min were provided in between each condition.

### Human-in-the-loop optimization (HIL optimization)

This protocol was provided to search the user’s optimal stiffness parameters of the exoskeleton for the descent and ascent phases of the squat using metabolic cost measured in real-time. We used the HIL Bayesian optimization method, which involves calculating the posterior distribution using measured metabolic cost given a parameter set and then selecting the next query parameters (ascending and descending squat stiffnesses (Fig. 1B)). We used the Gaussian process and Expected improvement for each process^69,70^. The selected ascending and descending stiffness parameters were used to provide assistance during the next iteration of squatting. This procedure was repeated until the stiffness parameters converged three times in a row, which took 15.8 ± 0.1 min. After the HIL optimization step, we let subjects rest for 45–54 min. The details can be found in Kantharaju et al.^55^.

### Validation study

The optimal stiffness condition determined from the HIL optimization procedure was compared with the no-device and unpowered conditions in the validation study. For each validation trial, subjects performed alternate squatting and standing movements for 4 min (Fig. 1C). Each squat cycle lasted 2 s (descending motion for 1 s, ascending motion for 1 s), and 6 s of rest in standing position. The validation study procedure is described in Kantharaju et al.^55^. The last 3 min of the collected data were considered for the analysis. Between each condition, the subjects rested for 12 min on a chair.

### Metabolic cost measurement

All participants wore the respiratory measurement device (K5, Cosmed, Rome, Italy). The Phase-plane based model-free estimation method^71^ was used to estimate the metabolic cost from the measured respiratory data. The details of metabolic cost measurement were described in Kantharaju et al.^55^.

### Torque assistance

In the ankle–foot exoskeleton, the torque assistance profile was divided into descending and ascending squat phases (Fig. 1B). Each torque profile was determined by multiplying each descending and ascending stiffness parameter by the ankle joint angle. The stiffness parameters were optimized for each participant or personalized using the HIL optimization. The torque profile using the personalized stiffness parameters presented high inter-subject variability shown by the high standard deviation (torque: 30.92 ± 8.68 Nm, torque by weight: 0.393 ± 0.094 Nm/kg (mean ± std)). The detailed control strategy is explained in Kantharaju et al.^55^.

### Muscle activation measurement

For each squatting condition, the muscle activation patterns were measured by EMG sensors (Trigno, Delsys Inc.). Eight EMG sensors were attached to the following muscles on the leg with the exoskeleton: soleus (SOL), gastrocnemius medialis (GASM), rectus femoris (RF), tibialis anterior (TA), vastus medialis and lateralis (VM, VL), bicep femoris (BF), and semitendinosus (ST)^43,44,72^. The electrodes were attached at the beginning of the study by following the instructions on “www.Seniam.org”^73^. The attached electrodes were wrapped with muscle tape to prevent them from getting detached. EMG data were collected at a sampling rate of 1259 Hz along with IMU data. The EMG signals were processed using a bandpass filter over the frequency range of 10–225 Hz (2nd order Butterworth), rectification, and lowpass filter at 10 Hz (2nd order Butterworth). The angular velocity was obtained from the IMU on the EMG sensor and was processed with a 5 Hz low pass filter. The squat phase (period of the onset, lowest position, and end of squat) was detected using the angular velocity in the mediolateral axis: At the onset of the squat, the angular velocity increases. At the lowest point of the squat, angular velocity returns to zero after the onset of the squat. At the onset of ascending squat, the angular velocity decrease. At the end of the squat, the angular velocity becomes zero. The identified squat phase was then used to extract the muscle activation signal from the EMG data. The extracted EMG data were normalized by the mean value of the unpowered condition from each subject^66,74^.

### Muscle synergy

For the fast computation of synergies, the processed EMG signals from the assisted leg were downsampled to 20 ms time bins^41^. EMG data were taken from the validation study, between 1 and 4 min. For the extraction of muscle synergies, the non-negative factorization (NNMF)^75,76^ method was adopted (MATLAB, settings: 1000 replicates), using (Eq. 1)

1 $$EM{G}_{n\times t}={W}_{n\times m}{H}_{m\times t}+{e}_{n\times t},$$

where EMG data is represented by the $n\times t$ matrix (n: number of EMG channels, t: length of sampled EMG data), and EMG data is decomposed into muscle synergy weight ${W}_{n\times m}$ , muscle synergy activation ${H}_{m\times t}$, and residual error matrices ${e}_{n\times t}$^24^.

For determining the proper number of synergies, the metric VAF (variance accounted for) was adopted^76^ using (Eq. 2).

2 $$VAF=1-\frac{{\sum }_{i=1}^{n}{\sum }_{j=1}^{t}{\left(EMG-{EMG}_{est}\right)}_{i,j}^{2} }{{\sum }_{i=1}^{n}{\sum }_{j=1}^{t}{\left(EMG-\overline{\mathrm{EMG} }\right)}_{i,j}^{2}}=1-\frac{{\sum }_{i=1}^{n}{\sum }_{j=1}^{t}{\left(EMG-{W}_{adj}{H}_{adj}\right)}_{i,j}^{2} }{{\sum }_{i=1}^{n}{\sum }_{j=1}^{t}{\left(EMG-\overline{\mathrm{EMG} }\right)}_{i,j}^{2}},$$

where *EMG* and $\overline{EMG }$ are the EMG signals and their mean values, i and j represent the i-th subject and j-th condition, and ${W}_{adj}$ and ${H}_{adj}$ are the length normalized synergy weights and activation. This method compares the sum of squared errors to the total squared sum of the EMG data. VAF values were extracted from each condition of each subject. While increasing the number of synergies from 1 to 7, if the minimum value among VAFs from the same subject was over 90%^76^, then the corresponding number of synergies was chosen for the subject.

### Synergy adjustment by data length

We used the NNMF algorithm (MATLAB), which automatically adjusts the RMS value of synergy activation to 1 regardless of signal data length. Hence, we normalized synergy activation and synergy weight matrix using (Eq. 3),

3 $${W}_{adj}=\frac{W}{\sqrt{n}}, {H}_{adj}=H\sqrt{n},$$

where $W, H$ are the muscle synergy weight and activation matrices, $n$ is the data length of the muscle synergy activation we investigated, ${W}_{adj},$ and ${H}_{adj}$ are the adjusted muscle synergy weight and activation.

### Synergy activation normalization

To compare synergy activations between conditions, we normalized synergy activation using (Eq. 4)^41^.

4 $${H}_{norm}=\frac{{H}_{adj}}{RMS\left({W}_{adj}\right)},$$

where ${H}_{adj}, {W}_{adj}$ are the adjusted muscle synergy weight and activation matrices. ${H}_{norm}$ is the normalized muscle synergy activation.

### Synergy similarity

A similarity index was used to classify synergy and to identify common strategies. For the similarity index, we have used cosine similarity^42^ (Eq. 5)

5 $$SI=\frac{{A}^{T}\cdot B}{\left|A\right|\cdot \left|B\right|},$$

where A and B are vectors with $m$ data. $SI$ has a range from − 1 to 1. The value 1 indicates that the directions of the vector A and B are similar, whereas the value − 1 indicates that the vectors are in the opposite directions. The value 0 indicates perpendicularity of vectors (no similarity). The threshold value of 0.8 is used to define the similarity between vector A and B^42^.

### Synergy classification

K-Means algorithm with cosine similarity was used to identify main synergy references and classify synergies. The K-means is an iterative algorithm that partitions the dataset^39,77^. First, the K-means extracted four synergy references, as most participants had four synergies (Table 1A). Then, the procedure compared subjects’ synergies with the synergy references. Specifically, a synergy with the highest cosine similarity with the reference group was assigned to its corresponding group^42^ with a no-duplication rule. In this classification, if the cosine similarity was below 0.6^42^, the synergy was assigned to the subject-specific synergy group.

### Statistical analysis

Statistical analyses were performed using R. We first checked the normality using the Shapiro–Wilk normality test^78^. We first investigated whether different conditions affected each outcome using repeated measures ANOVA with significance level α = 0.05. If significant effects were found, we compared all pairwise comparisons with a paired t-test using a Bonferroni-holm post hoc correction for multiple comparisons. If the normality test was not passed (Shapiro–Wilk test), we ran the Friedman test, and if significant, Wilcoxon signed rank test as a post hoc analysis with the Bonferroni-holm post hoc correction^79,80^. We excluded subject6’s data because the unpowered condition data was missing^81^.

### Correlation between muscle analysis and metabolic cost

We performed Pearson’s correlation analysis^45,64,82^ between the metabolic cost and the activation level of muscle synergy and RF muscle. Before conducting the correlation analysis, we normalized the metabolic cost and the muscle activation-related data from 0 to 1.

## Data Availability

The datasets generated during and/or analyzed during the current study are available from the corresponding author on reasonable request.
