# Author Correction: Muscle coordination and recruitment during squat assistance using a robotic ankle–foot exoskeleton

**DOI:** 10.1038/s41598-023-29611-y

**Published:** 2023-02-13

**Authors:** Hyeongkeun Jeong, Parian Haghighat, Prakyath Kantharaju, Michael Jacobson, Heejin Jeong, Myunghee Kim

**Affiliations:** 1 Department of Mechanical and Industrial Engineering, University of Illinois at Chicago, Chicago, IL 60607 USA; 2 Ira A. Fulton Schools of Engineering, Arizona State University, Arizona, Mesa, AZ 85212 USA

Correction to: *Scientific Reports* https://doi.org/10.1038/s41598-023-28229-4, published online 24 January 2023

The original version of this Article omitted an affiliation for the author Heejin Jeong.

The correct affiliations are listed below:

Department of Mechanical and Industrial Engineering, University of Illinois at Chicago, Chicago, IL, 60607, USA

Ira A. Fulton Schools of Engineering, Arizona State University, Arizona, Mesa, AZ, 85212, USA

The original Article has been corrected.

